# Changes in Behaviors and Attitudes in Response to COVID-19 Pandemic and Vaccination in Healthcare Workers and University Students in Italy

**DOI:** 10.3390/vaccines9111276

**Published:** 2021-11-03

**Authors:** Giorgia Della Polla, Concetta Paola Pelullo, Gabriella Di Giuseppe, Italo Francesco Angelillo

**Affiliations:** 1 Health Direction, Teaching Hospital, University of Campania “Luigi Vanvitelli”, Via Santa Maria di Costantinopoli 104, 80138 Naples, Italy; giorgia.dellapolla@unicampania.it; 2 Department of Experimental Medicine, University of Campania “Luigi Vanvitelli”, Via Luciano Armanni 5, 80138 Naples, Italy; concettapaola.pelullo@unicampania.it (C.P.P.); gabriella.digiuseppe@unicampania.it (G.D.G.)

**Keywords:** attitudes, behaviors, COVID-19, healthcare workers, Italy, students, vaccination, willingness

## Abstract

The objectives of the cross-sectional study were to measure how behaviors and attitudes about preventive measures toward COVID-19 changed over time among Italian vaccinated healthcare workers and university students, and the associated characteristics. The study was carried out between February and March 2021 in the city of Naples, Campania region, Southern Italy. The perceived personal risk of being infected by SARS-CoV-2 after the vaccination was significantly higher among males, in those having a higher perceived personal risk of being infected by SARS-CoV-2 before the vaccination, and in those who were more concerned about the efficacy of the vaccination. The fear of getting the disease as reason to have the COVID-19 vaccination was reported more frequently in younger participants, in those with at least one chronic medical condition, in those with a higher concern about the severity of COVID-19, in those with a higher level of trust in the information received, and in those who acquired information from scientific journals. Overall, 21.3% were willing to engage the three main public health measures (wearing a mask, careful hand washing, physical distancing) after receiving the second dose of the vaccination compared to the behavior before the pandemic began. This willingness was predicted by a higher level of trust in the information received and by a lower self-rated health status. Only 0.1% of participants were willing to engage all three measures after receiving the second dose of the vaccination compared to the behavior before receiving the first dose. These findings are useful in order to develop information strategies regarding vaccine safety and efficacy and the importance of public health measures against COVID-19.

## 1. Introduction

In Italy, after the declaration by the World Health Organization (WHO) that COVID-19 was a public health emergency on 20 January 2020 [1], the first official case of SARS-CoV-2 was detected on 21 February 2020. On 9 March 2020, the government announced a state of emergency, with numerous public health or restrictive policies to control the spread of the infection. Indeed, a general national lockdown had been imposed, with the closure of schools, universities, hospitality, bars, restaurants, all but essential retail, accompanied by the mandatory use of face masks in public areas, guidelines regarding social distancing, and washing hands regularly [2]. On 11 March 2020, the WHO declared the COVID-19 outbreak a global pandemic [3]. In autumn 2020, a second wave of the infection started in Italy, as in many other countries. To date, Italy is one of the worst-affected countries in Europe, with more than 4.5 million confirmed cases and 130,000 related deaths. The Campania region is the third most hit region in the country, with more than 452,000 total number of cases and 7800 deaths [4].

In Italy, the COVID-19 vaccination campaign was launched, as in many other countries, at the end of December 2020, among healthcare workers (HCWs) and residents of nursing homes, followed by elderly people, essential service providers, and people with chronic diseases. It is well known that no data were available at the time of this study regarding the duration of the protective effect and, therefore, some may be engaged in unprotected practices despite the frequent information provided during the pandemic period. These findings raise the need for a more cautious approach among those who completed the vaccination against COVID-19. Moreover, it is expected that people vaccinated would be more inclined to change their lifestyle and prevention attitudes and behaviors due to the optimism about the high efficacy of the vaccine. To the best of our knowledge, no studies have discussed this aspect in Italy. Thus, the interest was in examining whether receiving the vaccination against COVID-19 had an influence on an individual’s behaviors and attitudes regarding preventive measures. Specifically, the objectives of this study were to gain knowledge regarding (1) how behaviors and potential attitudes about preventive measures toward COVID-19 changed in Italy over time among vaccinated individuals and (2) the association between different characteristics of the respondents with their changes regarding behaviors and attitudes.

## 2. Materials and Methods

### 2.1. Study Design, Participants, and Sampling Procedures

The present study was part of several cross-sectional surveys conducted in the same geographic area during the outbreak of COVID-19, the first three of which have been previously published [5,6,7].

The study was carried out between February and March 2021 in the city of Naples, Campania region, in the Southern part of Italy. The study population comprised all students from a public university and all HCWs working in both clinical and nonclinical roles in a teaching hospital who attended the immunization center to receive the second dose of the COVID-19 vaccine.

The sample size was calculated considering that 50% of respondents changed their habits regarding wearing a mask, hand washing, and physical distancing after the second dose of the vaccination compared to the pre-pandemic period, using a confidence level of 95% and a margin of error of 5%. This returns a sample size of 385. In addition, this estimated size was adjusted for a non-response rate of 20%, yielding a final target sample population of 481 subjects.

### 2.2. Data Collection

The investigators approached all individuals attending the immunization center to solicit their interest and consent to join the survey after having explained the purposes and importance of the study. Prior to administering the survey, the investigators informed the participants that participation was voluntary, that the information collected was anonymous and confidential, and that no data could help to identify the responder. After such explanation, they were invited to participate in the study. Those who signed an informed consent received a hard copy of the questionnaire and instructions for the self-administration. In addition, they were also informed of their right to refuse to participate or to withdraw from the study, whenever they chose, without reprisal. No time limit was imposed on the participants. No compensation or incentive was given to the participants.

### 2.3. Study Tool

Information was collected through an anonymous self-administered questionnaire. A four-part questionnaire was prepared. The first part comprised several characteristics of the participants such as age, sex, educational level, professional role, working activity, underlying chronic medical conditions, infection with COVID-19, and self-rated health status. The second part focused on the attitudes and general concerns regarding COVID-19 and the vaccination. Participants were asked to rate their agreement to six perceived statements, including severity of the disease, level of trust in the information received on the vaccination, fear of becoming infected before and after the vaccination, and concern about safety and efficacy of the vaccine, measured with a ten-point Likert-type scale ranging from 1 = not at all to 10 = very considerably. The third part collected information on nine participants’ personal habits. Respondents were asked to report their behavior for each habit before the pandemic began (until February 2020) and before receiving the first dose of the vaccination (February 2021), and their willingness to adopt such habits after receiving the second dose of the vaccination. Participants were given “yes” or “no” response options for the behavior and “yes” or “no” or “don’t know” for the willingness. A question was asked about their reason(s) for being vaccinated. In the final part, participants were asked to indicate their most trusted sources of information regarding COVID-19 vaccination and whether they were interested in receiving further information. The questionnaire was tested in a pilot study among a group of 50 participants to determine its comprehensibility, face validity, and estimated completion time, which led to some refining of a few items to enhance the questionnaire. The survey responses from these first 50 respondents were not included in the overall analyses.

### 2.4. Ethics

The study protocol, the data collection instrument, and the consent form were approved by the Ethics Committee of the Teaching Hospital of the University of Campania “Luigi Vanvitelli”.

### 2.5. Statistical Analysis

Descriptive statistics were used to analyze the participants’ characteristics and responses to the different questions. Means with standard deviations (SD) and median with interquartile range were used for all continuous variables, whereas frequencies were used for the categorical variables. Then, univariate analysis was applied by using chi-square test or Student’s t-test to assess association, respectively, of categorical and continuous variables with the outcomes of interest. Variables that attained a *p*-value of less than 0.25 in the univariate analysis were included in the respective multivariate linear and logistic regression models, and the significant level of the *p*-value for the inclusion and elimination of the variables in the models was set at 0.2 and 0.4, respectively. Multivariate regression analysis was conducted to examine which of the different characteristics were significantly related to these three main following outcomes of interest: perceived personal risk of being infected by SARS-CoV-2 after receiving the second dose of the vaccination (Model 1), self-reported reason that it was important to have the vaccination for fear of getting COVID-19 (Model 2), and willingness to engage in the three main public health measures (wearing a mask, careful hand washing, physical distancing) after receiving the second dose of the vaccination compared to the behavior before the pandemic began (Model 3). The following selected predictor variables were included in all regression models: sex (female = 0; male = 1), age, in years (continuous), marital status (unmarried/separated/divorced/widowed = 0; married/cohabitant = 1), education level (undergraduate = 0; graduate = 1), having at least one chronic medical condition (no = 0; yes = 1), perception of personal health status (continuous), group (HCWs = 0; students = 1), trust in the information received on vaccination against COVID-19 (continuous), perceived personal risk of being infected by SARS-CoV-2 before the vaccination against COVID-19 (continuous), concern about the severity of COVID-19 (continuous), concern about the efficacy of the vaccine against COVID-19 (continuous), concern about the safety of the vaccine against COVID-19 (continuous), use of scientific journals as source of information about vaccination against COVID-19 (no = 0; yes = 1), and need of additional information regarding the vaccination against COVID-19 (no = 0; yes = 1). Moreover, the variable perceived personal risk of being infected by SARS-CoV-2 after the vaccination (continuous) was included in Model 3. In order to interpret the final multivariate regression models, odds ratios (OR) and their corresponding 95% confidence intervals (CI) were provided for the logistic analysis and ß coefficients and their standard errors for the linear analysis. For all analyses, *p*-values of less than 0.05 were considered statistically significant. The data collected were analyzed using the STATA 15.1 software [8].

## 3. Results

### 3.1. Characteristics of the Sample

Of the 1300 individuals that have been approached to participate in the study, 991 of them consented, giving a response rate of 76.2%. Table 1 summarized the principal characteristics of the sample. Most females completed the questionnaire, the median age was 23 years, the vast majority were unmarried, only 7.8% had at least one chronic medical condition, only 5.8% reported having tested COVID-19 positive, and only 7.2% self-rated that their health condition had worsened compared to a year ago.

### 3.2. Beliefs and Attitudes

The details of the participants’ beliefs and attitudes towards COVID-19 and its vaccination, measured on a 10-point Likert-type scale, are showed in Table 2. Almost one fourth (22.1%) were very concerned about the severity of COVID-19, with an overall average value of 7.9. The perceived personal risk of being infected by SARS-CoV-2 resulted with mean values of 6.9 and 4.3 before and after the vaccination, respectively. Regarding the statements of the perceived risk of the vaccination against COVID-19, the mean total values of the concern about its efficacy and safety were respectively 4.1 and 4.5, which indicate a low perceived level of concern. Overall, only 2.4% and 12.8% of the respondents revealed high concern about the efficacy and safety, whereas, respectively, 18.9% and 3.5% expressed no concern at all. Finally, around one-fifth (19.6%) of the sample displayed a high trust in the information received on the vaccination against COVID-19 and only 0.9% did not trust it at all, with an overall mean value of 7.8.

To better understand the reasoning behind having the vaccine against COVID-19, it was asked why they had taken it. To protect their family members (81.5%) was the main self-declared reasoning, followed by the protection for themselves (63.1%), the efficacy (46.7%) and the safety (41.7%) of the vaccine, and the fear of getting the disease (40.1%).

The details of the participants’ behavior regarding different habits before the pandemic began (until February 2020) and before receiving the first dose of the vaccination (February 2021), and their willingness toward such habits after receiving the second dose of the vaccination, are presented in Table 3.

There was a marked increase in the frequency of respondents who have changed their behaviors and have adopted all three main public health measures (wearing a mask, careful hand washing, physical distancing) to protect themselves before receiving the first dose of the vaccination compared to the behaviors before the pandemic began, with values ranging from 21% to 94.3% for the mask, 30.4% to 94.6% for the physical distancing, and 70.5% to 95.1% for hand washing. Overall, 21.3% of the respondents were willing to engage in the three measures after receiving the second dose of the vaccination compared to the behavior before the pandemic began. A slightly not significant decrease has been observed regarding the willingness toward maintaining physical distancing and hand washing after receiving the second dose of the vaccination compared to the behaviors before the first dose, whereas no change has been observed for wearing a mask (data not shown). Overall, only 0.1% of participants were willing to engage in the three measures after receiving the second dose of the vaccination compared to the behavior before the first dose.

### 3.3. Predictors of the Attitudes

The results of the multivariate linear and logistic regression models predicting the different outcomes of interest are summarized in Table 4. Stepwise multivariate linear regression analysis showed that males, those who perceived a higher personal risk of being infected by SARS-CoV-2 before the vaccination, and those who were more concerned about the efficacy of the vaccine against COVID-19 were more likely to perceive a higher level of personal risk of being infected by SARS-CoV-2 after receiving the second dose of the vaccination (Model 1).

The results of the multivariate logistic regression model of the factors associated with the self-reported reason that it was important to have the COVID-19 vaccination for fear of getting the disease revealed that this reason was reported more frequently by participants of a younger age (OR = 0.97; 95% CI = 0.94–0.99) with the odds decreasing by 0.97 times per year of age, those with at least one chronic medical condition (OR = 2.25; 95% CI = 1.28–3.94), those who were more concerned about the severity of COVID-19 (OR = 1.21; 95% CI = 1.1–1.34), those with a higher level of trust in the information received on the vaccination against COVID-19 (OR = 1.18; 95% CI = 1.08–1.29), and those who had used scientific journals as a source of information about vaccination against COVID-19 (OR = 1.77; 95% CI = 1.31–2.39) (Model 2).

The multivariate logistic regression analysis revealed that having a higher level of trust in the information received on the vaccination against COVID-19 (OR = 1.11; 95% CI = 1.01–1.23) and a lower self-rated health status (OR = 0.66; 95% CI = 0.5–0.87) predicted the willingness to engage the three main public health measures (wearing a mask, careful hand washing, physical distancing) after receiving the second dose of the vaccination compared to the behavior before the pandemic began (Model 3).

### 3.4. Sources of Information

All respondents (99.8%) stated that they had received information about the vaccination against COVID-19. From the multiple-selection question related to specific sources of information, it was revealed that the most trusted source was the Internet (51.3%), followed by newspaper, TV and radio (49.3%), and scientific journals (45.5%), while health care professionals was listed by only 42.7% of the respondents. More than one-third of the sample (38.7%) wished to receive additional information.

## 4. Discussion

As far as we know, there are no other similar cross-sectional surveys conducted in Italy, and this study provides information on changes in behaviors and on potential attitudes about several widely accepted preventive measures toward COVID-19 from the pre-pandemic period among vaccinated individuals.

One of the most interesting findings was the considerable change in the respondents’ self-reported adoption of the three main public health measures (wearing a mask, careful hand washing, physical distancing) before the pandemic began (until February 2020), before receiving the first dose of the vaccination (February 2021), and their willingness towards such measures after receiving the second dose. Indeed, one out of five respondents were willing to engage in these three measures after receiving the second dose of the vaccination compared to the behavior before the pandemic began. A plausible interpretation of these changes is that the application of these measures prevents the spread of COVID-19 [9,10,11,12]. In addition, the willingness to engage in the three main public health measures after receiving the second dose of the vaccination did not differ compared to the behaviors before receiving the first dose. This result may be explained by the fact that respondents, although fully vaccinated, feel that they must continue to adopt these preventive measures since the results regarding the duration of the protective effect of the vaccine were not available yet.

The present group of participants mostly had positive attitudes and beliefs towards COVID-19 and its vaccination. The individuals’ perceived personal risk of being infected by SARS-CoV-2 after the vaccination resulted with a mean value of 4.3. It is interesting to note that, in the multivariate linear regression analysis, the higher perceived personal risk of being infected by SARS-CoV-2 before the vaccination and the higher concern about the efficacy of the vaccine significantly influenced a higher level of perceived personal risk of being infected after the vaccination. Health educational campaigns need to be conducted with the important objective being to clarify the concerns regarding efficacy of the vaccination. This is not surprising given the fact that the students represented the vast majority of the sample. The major concern regarding the perceived lack of efficacy reinforces previous reports on respondents who had doubts about the performance of the vaccine [13,14,15,16,17,18,19]. This large group of participants allowed to identify the most commonly reasons that contributed to respondents’ decisions on COVID-19 vaccination. The finding highlighted that the principal reason for accepting this vaccination was an altruistic motivation, with the desire to prevent the illness for their family members (81.5%) followed by the benefits to personal well-being (63.1%). This may partly be explained by the younger age and the unmarried status of the participants who live in the same household with members of an older age and, therefore, at higher risk of getting the disease. It should be underlined that only less than half stated its efficacy and safety (side effects) as reasons for vaccination. A possible explanation for this lower frequency may include the rapid pace of the COVID-19 vaccine development and the limited information available about the safety at the time of this data collection. This is consistent with the observation of a progressive increase in the individuals who would accept this vaccine as the vaccination time neared [20]. The finding in the present study may also reflect a lack of information since it should be noted that 38.7% of the respondents wished to receive additional information about this vaccination. Moreover, 40.1% indicated the fear of getting the disease as a reason for COVID-19 vaccine acceptance. Interestingly, the results of the multivariate logistic regression model revealed that having received this vaccination for fear of getting the disease was more likely to be indicated by the respondents of a younger age, those with at least one chronic medical condition, those more concerned about the severity of COVID-19, and those with a higher trust in the information received on the vaccination. The higher fear among those of a younger age parallels other observations in this geographic area [5] and worldwide [21,22,23]. A possible explanation is that younger people are more likely to spend time socializing with others and to be engaged in pleasant activities and, therefore, they perceive to be at higher risk of getting the disease. Furthermore, the finding that participants with underlying comorbidities were over two times more likely to receive the vaccination for fear of getting COVID-19 is conceivable, with the fact that they interact more regularly with physicians and may therefore be more likely to receive a recommendation in order to preserve their health condition [24,25].

When respondents were asked about information sources regarding COVID-19 vaccination, more than half have used the Internet. This finding was expected since students were the main study group, and it is well known that the Internet has become an essential part of their daily life as a preferred source for searching and sharing information. However, this is worrying since there is a large amount of unreliable, unverified, and contradicting information that is not peer reviewed on Internet websites about vaccination and, therefore, such information should be managed with caution [26,27]. Moreover, an evolving body of literature showed that the use of the Internet has been consistently associated with a lower level of knowledge [28,29,30], less positive attitudes, and decreased use of preventive services with, for example, lower uptake and willingness to have the vaccination [31,32,33]. Therefore, it is an important priority to ensure information and communication strategies to improve the level of knowledge and the adoption of appropriate preventive behaviors towards COVID-19. It should be noted that scientific journals and health care professionals were mentioned as sources of information by less than half of the participants. These sources have an important role in accurate, clear, transparent information and communication. Indeed, having received information about COVID-19 vaccination from scientific journals was one of the most significant influential factors of the self-reported reason that it was important to have this vaccination for fear of getting the disease. This is consistent with previous studies also conducted in other countries that have shown that having received information from the scientific journals had a positive influence on the individual decision making regarding vaccination [29,34,35,36,37,38,39,40,41,42]. There is clearly a need for HCWs, as part of this sample, to be able to develop competencies aimed to overall improve the vaccination uptake for them and for the general population in this geographic area. For instance, 34.7% of parents were hesitant about the childhood vaccinations [43], only 15.3% of parents had immunized their child against rotavirus infection [44], only 16.9% have received at least one vaccination recommended to adults between 19-64 years of age [45], and 57.8% of parents had vaccinated their child aged 12-13 years against HPV [29].

The results from the present survey should also be considered with a few methodological limitations in mind. First, the cross-sectional nature of the survey; hence, only associations can be presented, and no conclusions can be drawn regarding the causal relationship between the determinant factors and the different outcomes of interest because temporal sequence cannot be established. Second, the survey was administered at a single center and, consequently, generalizability of the study may also be limited, and conclusions may not be representative and inferable to individuals in other regions of Italy. Third, the study findings are derived from self-reported data. It is possible that the results may have been affected by reporting bias and social desirability bias and, therefore, may not accurately reflect the behavior and the attitudes as respondents would select the answer preferred by the researchers. The potential overestimation of preventive measures compliance should be acknowledged. Notwithstanding the above-mentioned limitations, this survey provides insight into the individuals’ perceptions and health behaviors and willingness regarding preventive measures after receiving the vaccination against COVID-19.

## 5. Conclusions

In conclusion, the results of this survey make a significant contribution around the changes in the behaviors and potential attitudes on the adherence with COVID-19 pandemic preventive measures of vaccinated individuals and the role of potential explanatory variables. One out of five respondents were willing to engage the three main public health measures after receiving the second dose of the vaccination compared to the behavior before the pandemic began, and the majority demonstrated positive attitudes and beliefs towards COVID-19 and its vaccination. The survey suggests that there is an immediate need for the development of effective information strategies to answer concerns, mainly regarding the vaccines’ safety and efficacy and the importance of public health measures against COVID-19.

## Figures and Tables

**Table 1:** Respondents’ principal characteristics.

Characteristics	N	%
Age, years	23 (22–25.5) *	
Sex		
Female	641	65.2
Male	342	34.8
Marital status		
Unmarried/widowed/separated/divorced	896	93.3
Married/cohabitant	64	6.7
Education level		
High school degree or less	805	81.9
College degree or higher	178	18.1
Having at least one chronic medical condition		
No	898	92.2
Yes	76	7.8
Group		
Students	821	82.9
HCWs	170	17.1
Having tested COVID-19 positive		
No	924	94.2
Yes	57	5.8
Self-rated health status		
Better than one year ago	47	4.8
A little better than one year ago	86	8.9
More or less the same than one year ago	766	79.1
A little worse than one year ago	66	6.8
Worse than one year ago	4	0.4

* Median (interquartile range). The number of respondents to each question may not add up to total number of study population due to missing values.

**Table 2:** Respondents’ beliefs and attitudes towards COVID-19 and its vaccination.

Statements	Mean (SD)
Concern about the severity of COVID-19	7.9 (1.8)
Perceived personal risk of being infected by SARS-CoV-2 before the vaccination against COVID-19	6.9 (2.3)
Perceived personal risk of being infected by SARS-CoV-2 after the vaccination against COVID-19	4.3 (2.3)
Concern about the efficacy of the COVID-19 vaccination	4.1 (2.5)
Concern about the safety of the COVID-19 vaccination	4.5 (2.5)
Trust in the information received about COVID-19 vaccination	7.8 (1.9)

**Table 3:** Respondents’ behaviors regarding different habits before the pandemic began and before receiving the first dose of the vaccination, and willingness toward such habits after receiving the second dose of the vaccination.

Habits	Behavior before the Pandemic Began		Behavior before Receiving the First Dose of the Vaccination		Willingness after Receiving the Second Dose of the Vaccination	
	n	%	n	%	n	%
Wearing a mask	205	21	911	94.3	886	94.5
Careful hand washing	689	70.5	929	95.1	884	94.3
Maintain physical distancing	298	30.4	931	94.6	865	91.9
Doing physical activity outdoors	649	66.6	452	46.4	462	49.5
Eating at a restaurant	831	85.1	350	35.9	385	41.2
Using public transport	727	74.3	310	31.7	377	40.2
Attending indoor public places	778	80.3	201	20.7	210	22.7
Greeting with a handshake	793	81.1	82	8.4	97	10.3
Attending indoor and outdoor crowded places	702	71.6	68	6.9	85	9

The number of respondents to each item may be different due to missing values.

**Table 4:** Multivariate linear and logistic regression analysis results examining the outcomes of interest according to several explanatory variables.

Variable	Coeff.	SE	*t*	*p*
Model 1. **Perceived** **risk** **of being infected by SARS-CoV-2 after receiving the second dose of the vaccination (Sample size = 836)**				
*F* (7828) = 106.82, *p* < 0.0001, *R*^2^ = 47.4%, adjusted *R*^2^ = 47%				
Perceived personal risk of being infected by SARS-CoV-2 before the vaccination	0.18	0.03	5.8	<0.001
Concerned about the efficacy of the vaccine against COVID-19	0.6	0.02	24.19	<0.001
Sex				
Female	1.00 *			
Male	0.26	0.12	2.08	0.038
Group				
Students	1.00 *			
HCWs	0.31	0.19	1.6	0.11
Age	0.02	0.01	1.43	0.152
Concerned about the severity of COVID-19	−0.05	0.04	−1.42	0.157
At least one chronic medical condition				
No	1.00 *			
Yes	0.27	0.22	1.19	0.234
	OR	SE	95% CI	*p*
Model 2. **Self-reported reason that it was important to have the COVID-19 vaccination for fear of getting the disease (Sample size = 828)**				
Log likelihood = −519.22, χ^2^ = 79.85 (8 df), *p* < 0.0001				
Concerned about the severity of COVID-19	1.21	0.06	1.1–1.34	<0.001
Level of trust in the information received on the vaccination against COVID-19	1.18	0.05	1.08–1.29	<0.001
Scientific journals as source of information about vaccination against COVID-19				
No	1.00 *			
Yes	1.77	0.27	1.31–2.39	<0.001
At least one chronic medical condition				
No	1.00 *			
Yes	2.25	0.64	1.28–3.94	0.005
Age	0.97	0.01	0.94–0.99	0.02
Self-rated health status	1.27	0.17	0.98–1.66	0.074
Concerned about the safety of the vaccine against COVID-19	0.95	0.03	0.89–1.01	0.1
Sex				
Female	1.00 *			
Male	1.19	0.19	0.87–1.63	0.267
Model 3. **Willingness to engage in the three main public health measures (wearing a mask, careful hand washing, physical distancing) after receiving the second dose of the vaccination compared to the behavior before the pandemic began (Sample size = 843)**				
Log likelihood = −423.54, χ^2^ = 27.3 (4 df), *p <* 0.0001				
Self-rated health status	0.66	0.09	0.5–0.87	0.003
Level of trust in the information received on vaccination against COVID-19	1.11	0.05	1.01–1.23	0.03
Concerned about the safety of the vaccine against COVID-19	0.92	0.04	0.84–1.01	0.07
Concerned about the efficacy of the vaccine against COVID-19	0.94	0.04	0.86–1.03	0.21

* Reference category.

## Data Availability

The data presented in this study are available on request from the corresponding author.
